# Supplementary material for: Unique trajectory of gene family evolution from genomic analysis of nearly all known species in an ancient yeast lineage
Source: Mol Syst Biol. 2025 May 27;21(8):1066–89. doi: 10.1038/s44320-025-00118-0 (PMC12322030; doi:10.1038/s44320-025-00118-0)
Supplement: Supplementary file 1 — Appendix [file 44320_2025_118_MOESM1_ESM.pdf]

Appendix for:

Unique trajectory of gene family evolution from genomic analysis of nearly all known species in an ancient yeast lineage.

Table of Contents

Appendix Figure S1. Comparative analysis of evolutionary rates versus weighted average gene family size in Pichiales and Serinales……………………………………………………..3

Appendix Figure S2. DBSCAN clustering based on evolutionary rates (branch lengths)…….4

Appendix Figure S3. PCA of gene families with mean coverage larger than 0.1……………..5

Appendix Figure S4. Comparative analysis of PC1 coordinates between FELs and SELs….7

Appendix Figure S5. Identification of 27 gene families representing PC2 in the PCA with a 0.5 coverage threshold……………………………………………………………………………..8

Appendix Figure S6. Orphan gene families across 12 orders…………………………………...9

Appendix Figure S7. Origins of orphan genes…………………………………………………...10

Appendix Figure S8. Weighted average gene family size using different weighting methods………………………………………………………………………………………………11

Appendix Figure S9. Comparison of weighted average gene family size across yeasts, filamentous ascomycetes, animals, and plants…………………………………………………12

Appendix Figure S10. Average gene family copy number comparisons……………………...13

Appendix Figure S11. Schematic of Spearman rank correlation analysis…………………….14

Appendix Figure S12. PCA on copy number datasets…………………………………………15

Appendix Figure S13. PCoA on presence/absence dataset using Euclidean distance……..16

Appendix Figure S14. PCoA on presence/absence dataset using Manhattan distance…….17

Appendix Figure S15. PCoA on presence/absence dataset using Jaccard distance………..18

Appendix Figure S16. Functional enrichment analysis on PCoA1 using Jaccard and Euclidean results……………………………………………………………………………………19

Appendix Figure S17. Distribution of HGT genes in 0.1 and 0.5 coverage gene family datasets……………………………………………………………………………………………....20

Appendix Figure S18. Weighted average gene family size of 0.1 coverage dataset excluding HGT genes…………………………………………………………………………………………..21

Appendix Figure S19. Fold change PCA, and functional enrichment analyses excluding HGT genes……………………………………………………………………………………………22

Appendix Table S1. Correlations between PICs of weighted average gene family size with both gene number and genome size……………………………………………………………...23

Appendix Table S2. Multimodality analyses in weighted average size and evolutionary rate within each order……………………………………………………………………………….24

Appendix Table S3. Gene families that experienced significant contractions or losses in Saccharomycodales and Trigonopsidales, corresponding to Dipodascales………………….25

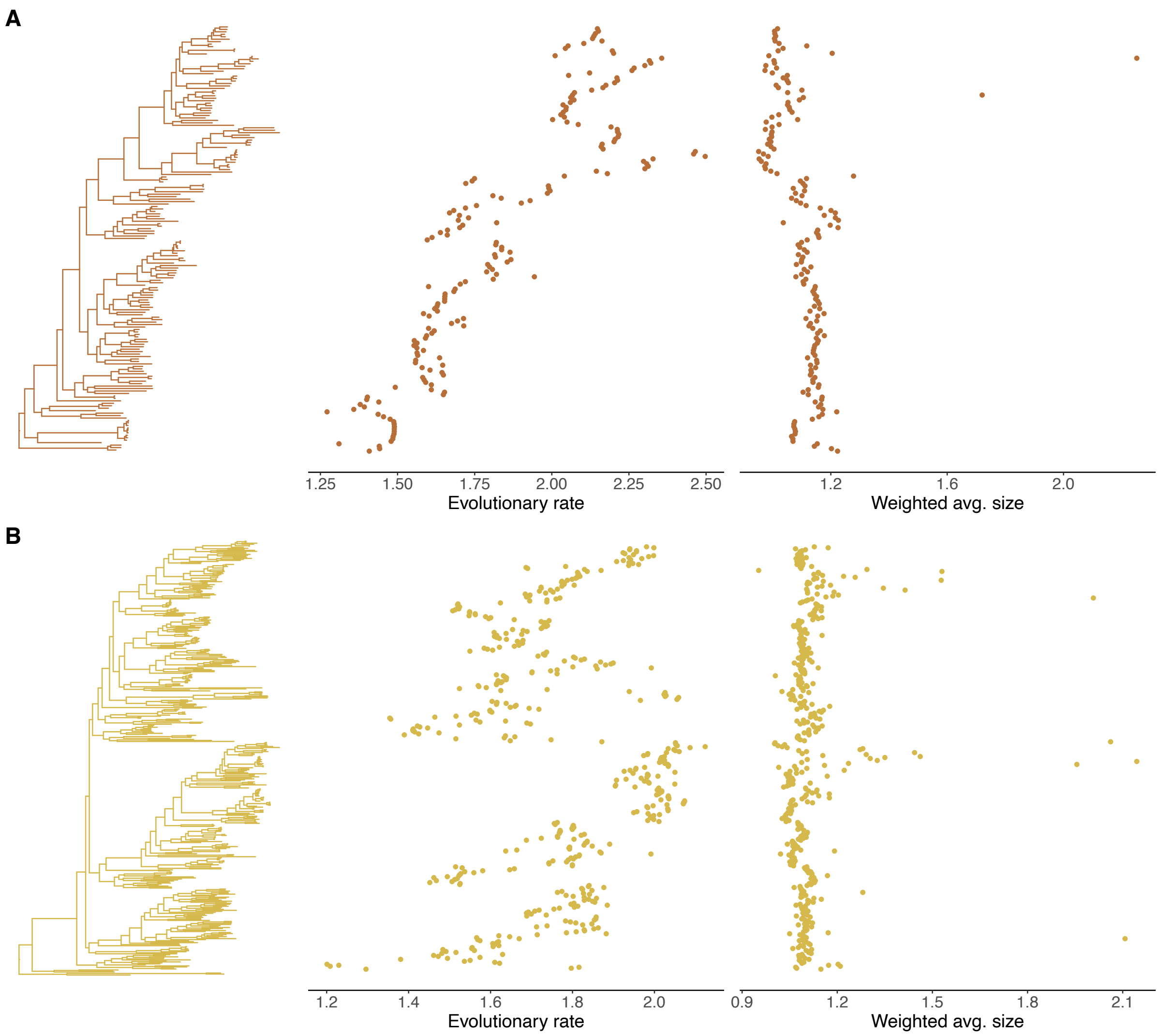

Appendix Figure S1 - Comparative analysis of evolutionary rates versus weighted average gene family size in Pichiales and Serinales.

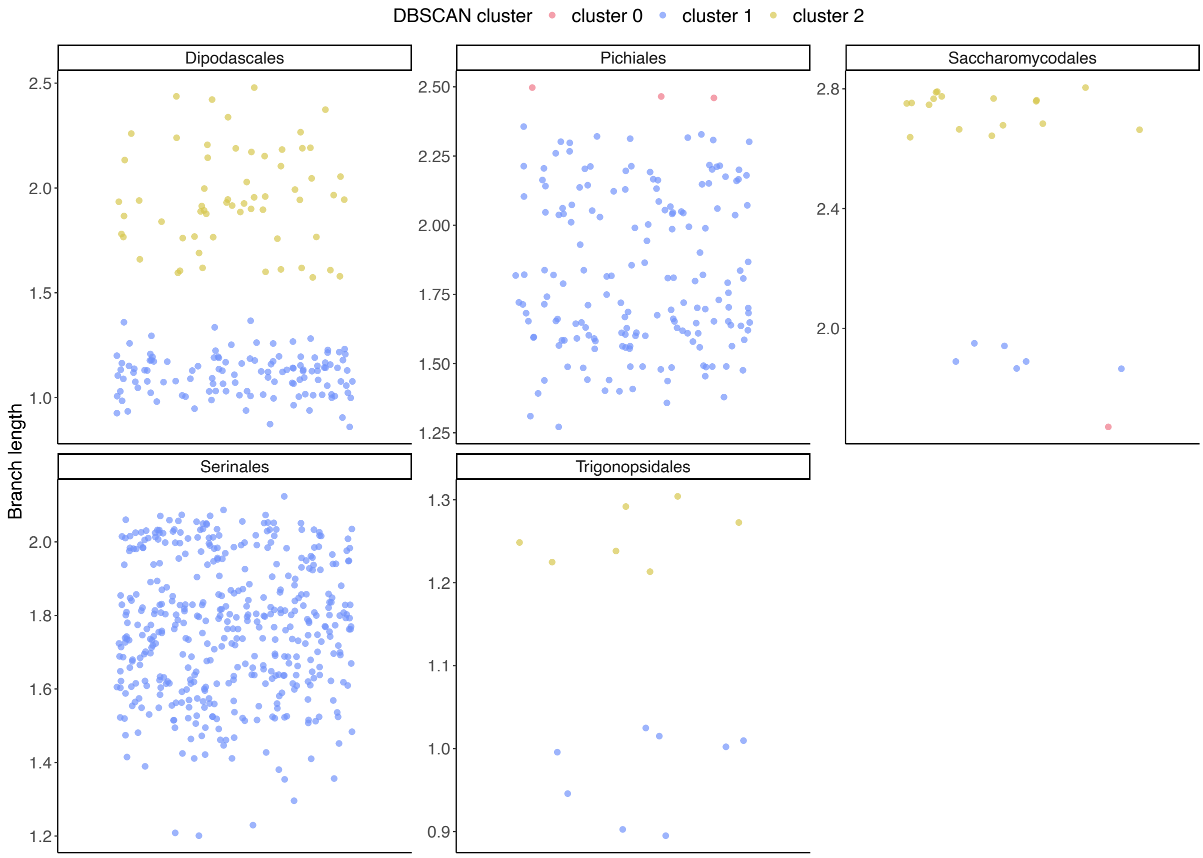

Appendix Figure S2 - DBSCAN clustering based on evolutionary rates (branch lengths).

In the DBSCAN clustering, cluster 0 is designated for noise points, but these will be assigned to the nearest cluster for analysis. In Dipodascales, Saccharomycodales, and Trigonopsidales, cluster 1 represents slower-evolving lineages, while cluster 2 represents faster-evolving lineages.

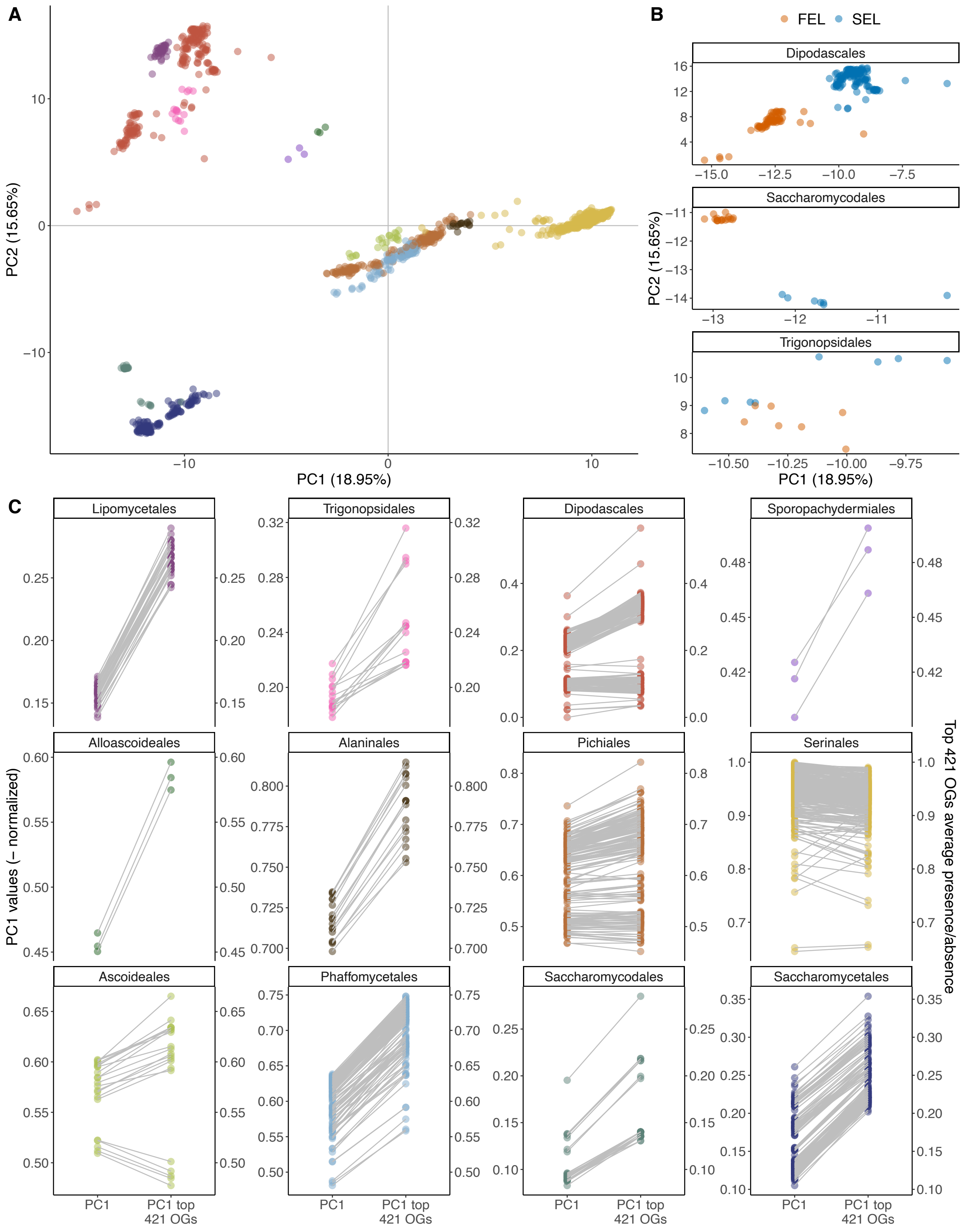

Appendix Figure S3 - PCA of gene families with mean coverage larger than 0.1.

A PCA analysis utilizing presence and absence data for 5,551 gene families with an average coverage of 0.1 or greater.

B Highlight the FEL and SEL within the Dipodascales, Saccharomycodales, and Trigonopsidales orders.

C Using the same method as in the PCA with a 0.5 coverage threshold, 421 gene families (with the highest absolute correlation (rho = 0.990)) were identified to represent Principal Component 1. PC1 coordinates were normalized. Points on the plot represent individual yeasts, with lines connecting the same yeast. No significant enrichment results were found for these 421 gene families.

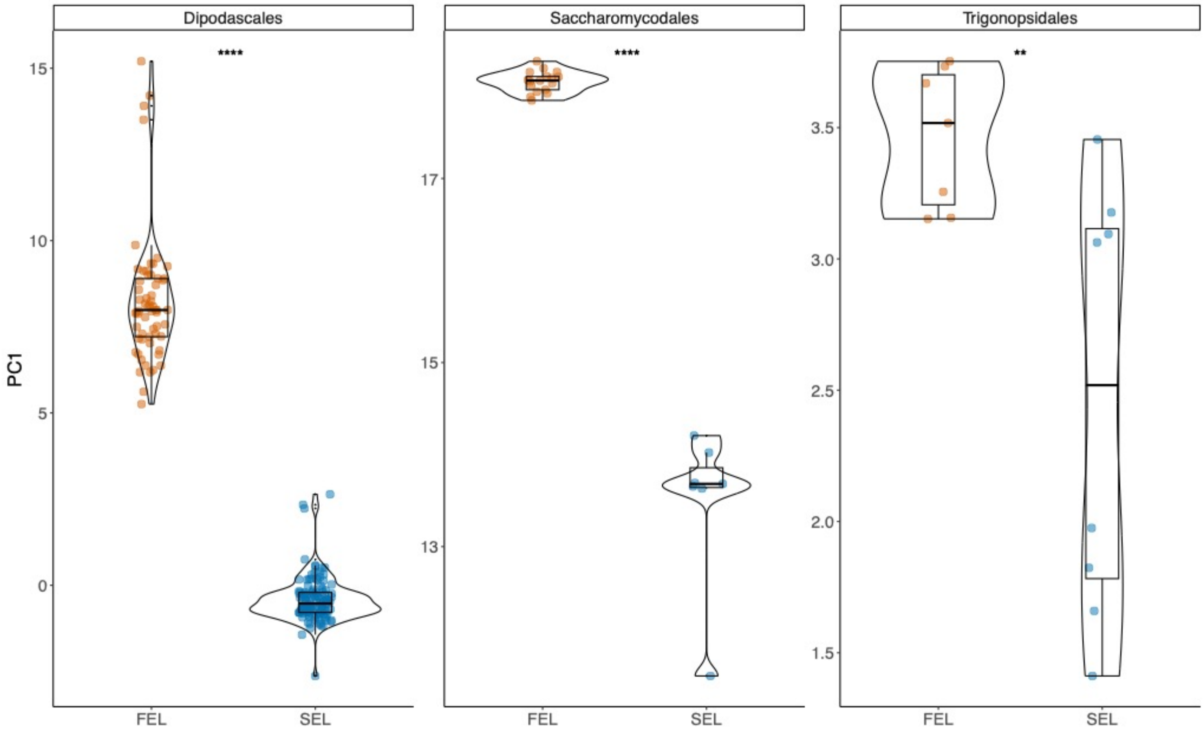

Appendix Figure S4 - Comparative analysis of PC1 coordinates between FELs and SELs.

PC1 coordinates were derived from the PCA with a 0.5 coverage threshold. Wilcoxon tests were used to determine significance, where “**” represents $P < 0.01$ and “****” indicates $P < 0.0001$.

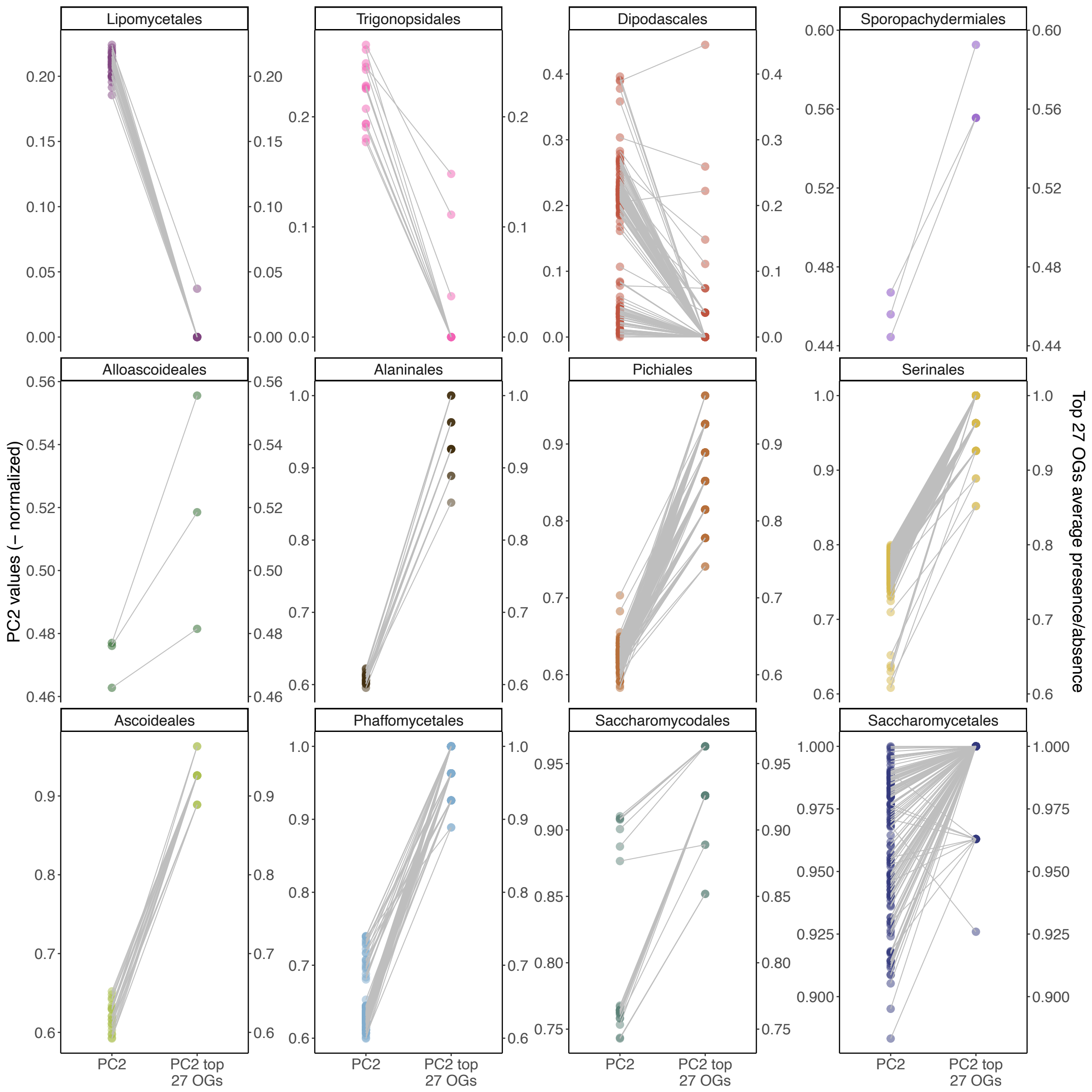

Appendix Figure S5 - Identification of 27 gene families representing PC2 in the PCA with a 0.5 coverage threshold.

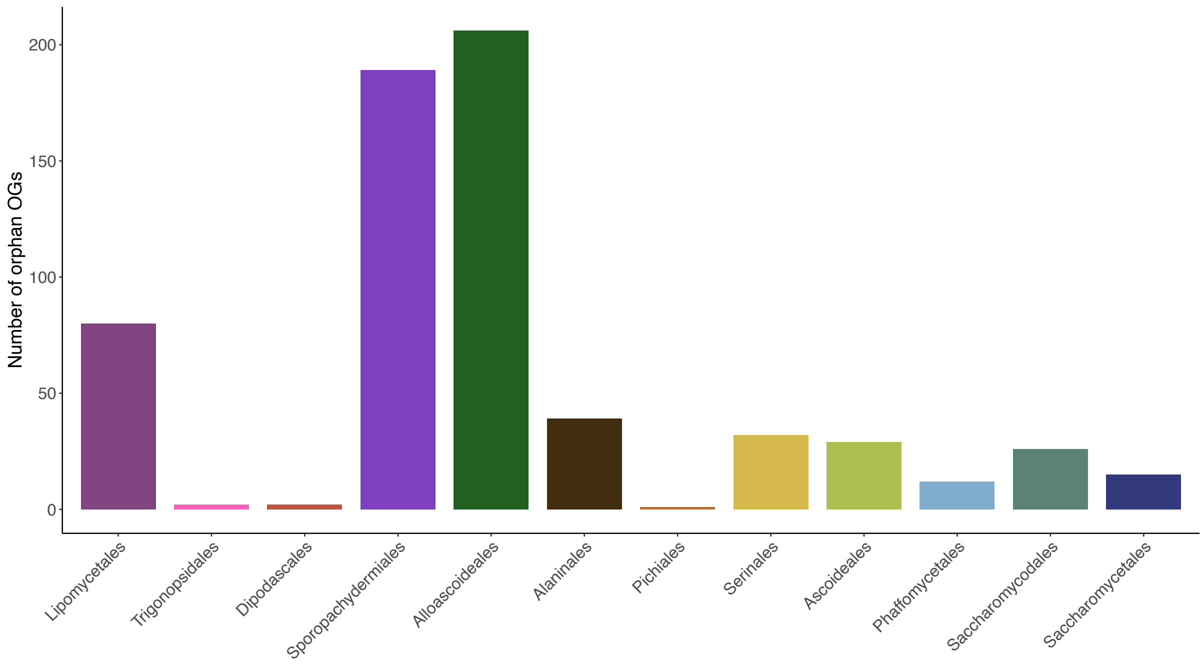

Appendix Figure S6 - Orphan gene families across 12 orders.

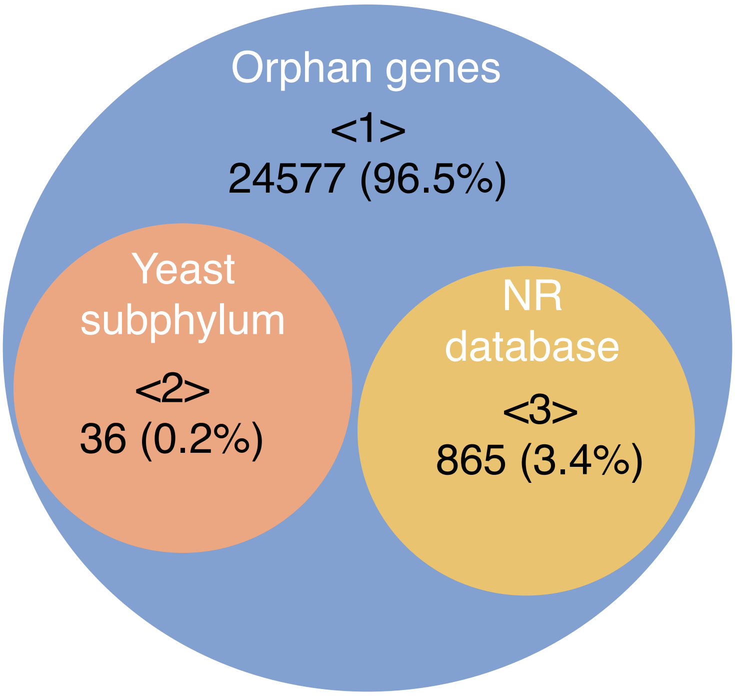

Appendix Figure S7 - Origins of orphan genes.

The blue circle (<1>) represents all orphan genes, the orange circle (<2>) represents orphan genes with protein homologs in other orders (excluding the order to which the specific orphan gene belongs), and the yellow circle (<3>) represents orphan genes with protein homologs in the NR database (excluding Saccharomycotina).

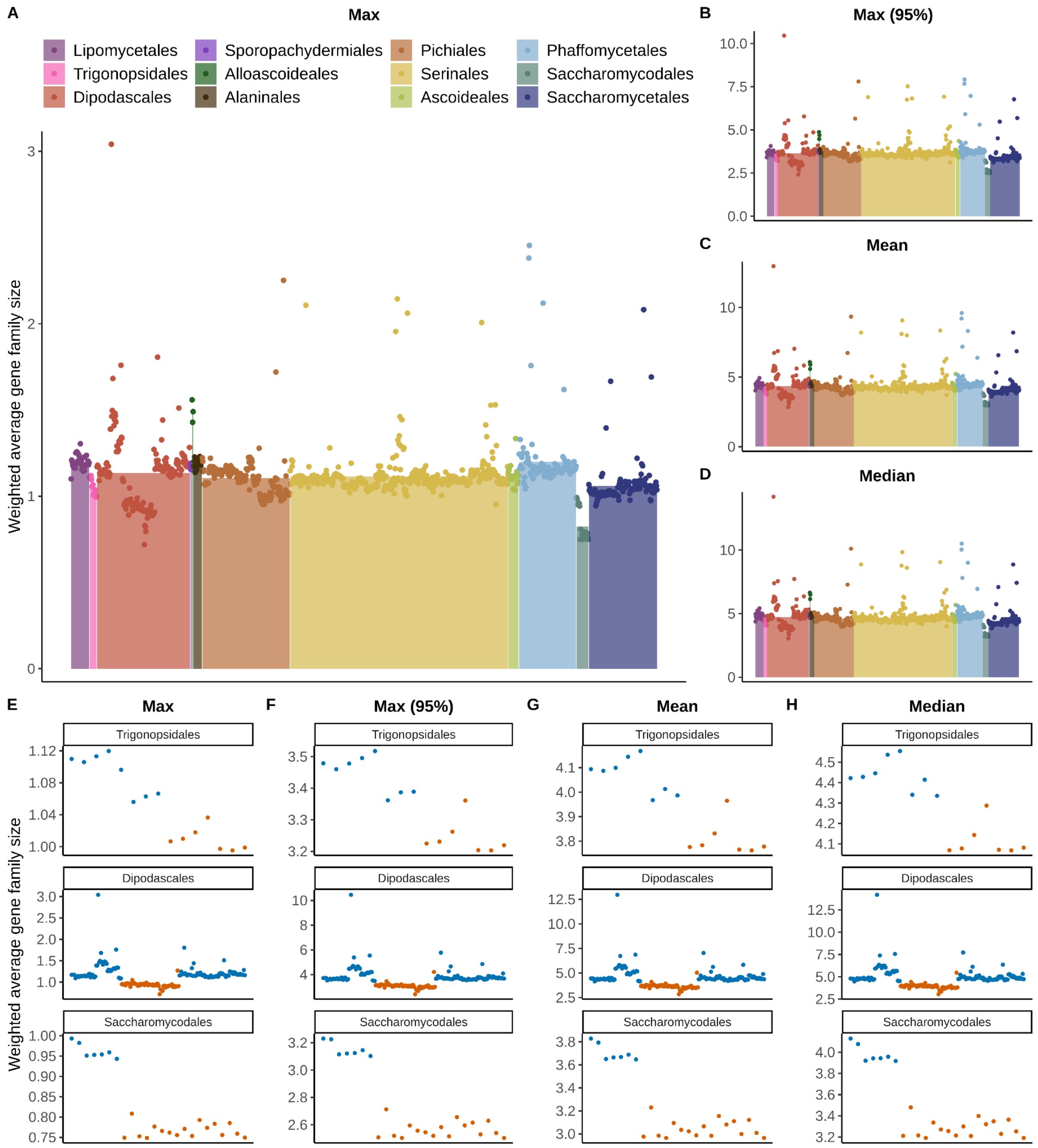

Appendix Figure S8 - Weighted average gene family size using different weighting methods.

The arrangement of yeasts on the x-axis follows the same order as in the phylogenetic tree and aligns with Figure EV2.

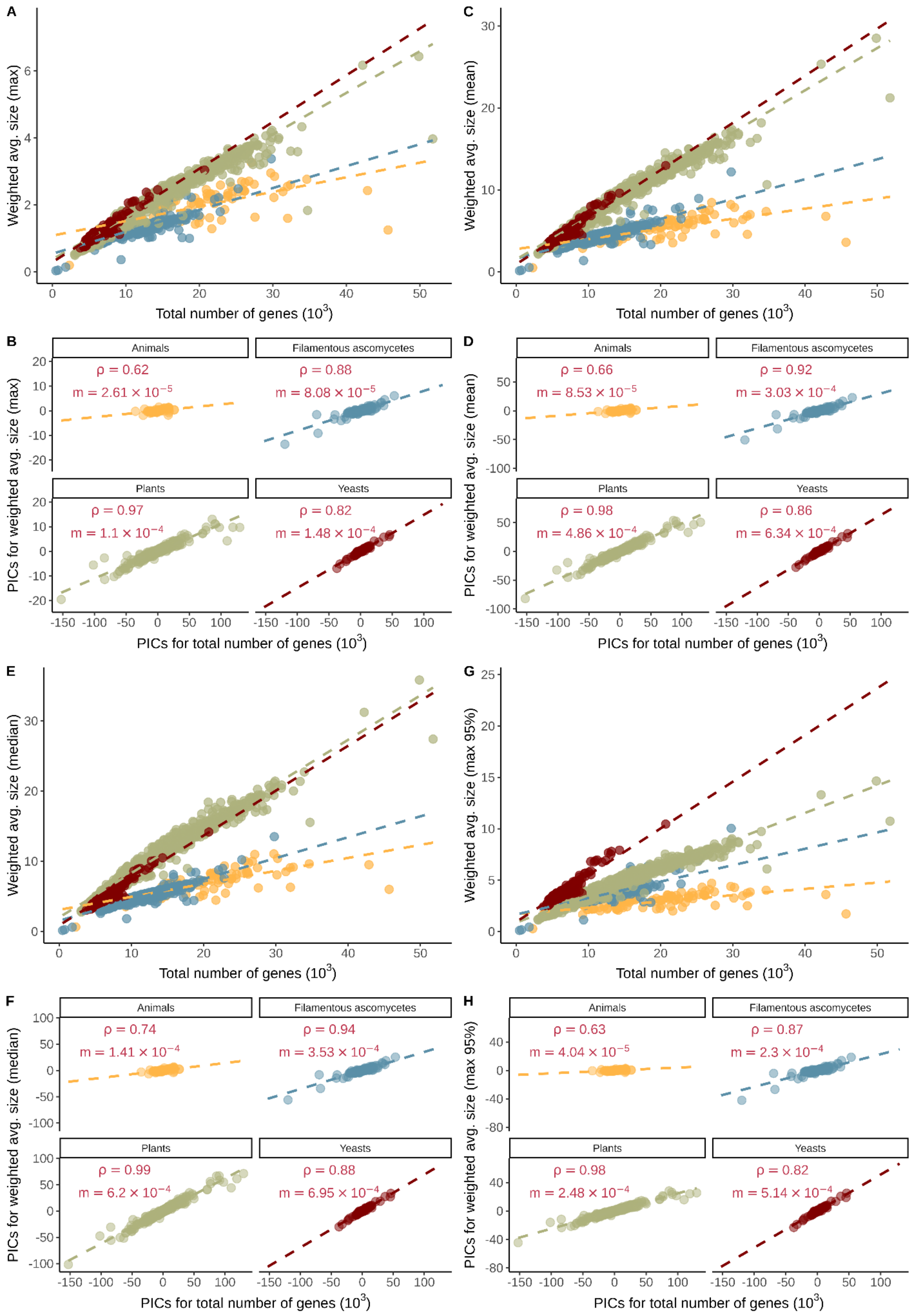

Appendix Figure S9 - Comparison of weighted average gene family size across yeasts, filamentous ascomycetes, animals, and plants.

Similar to Figure 1B and 1C in our manuscript, but generated using different weighting methods.

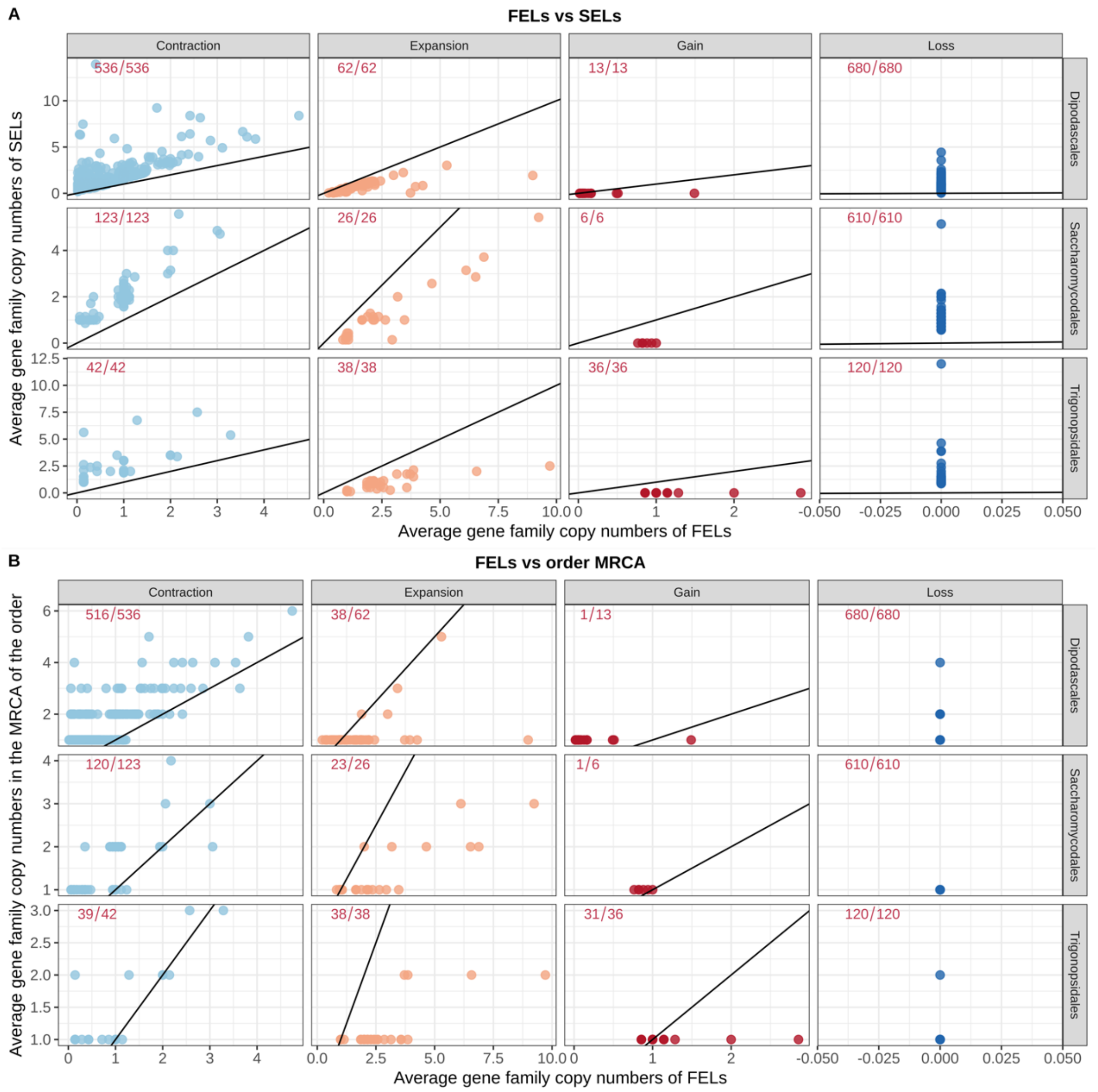

Appendix Figure S10 - Average gene family copy number comparisons.

This figure presents comparisons of average gene family copy numbers, focusing only on gene families identified as experiencing significant changes in the fold change analysis.

A Average gene family copy numbers of FELs compared to their respective SELs. The solid diagonal line represents equal values; points below the line indicate that FELs have larger average numbers than SELs. In the contraction/loss panels, annotations such as “536/536” and “13/13” indicate that 536 out of 536 and 13 out of 13 gene families, respectively, in FELs are smaller than in SELs.

B Average gene family copy numbers of FELs compared to the MRCA of their respective orders.

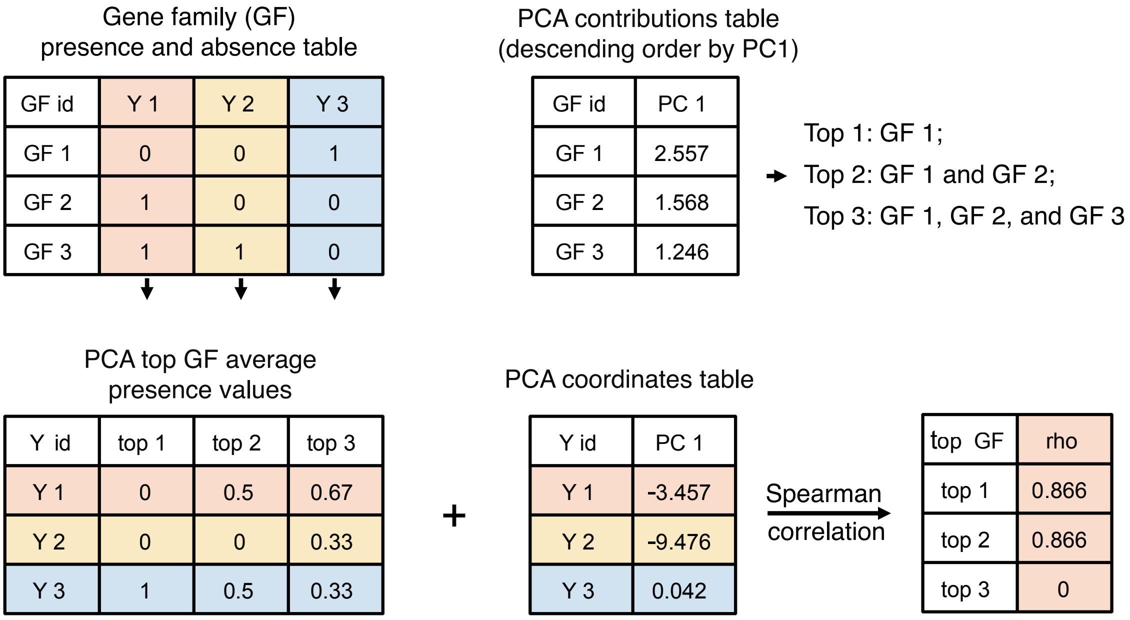

Appendix Figure S11 - Schematic of Spearman rank correlation analysis.

Y represents the specific yeast, and rho denotes the Spearman rank correlation coefficient.

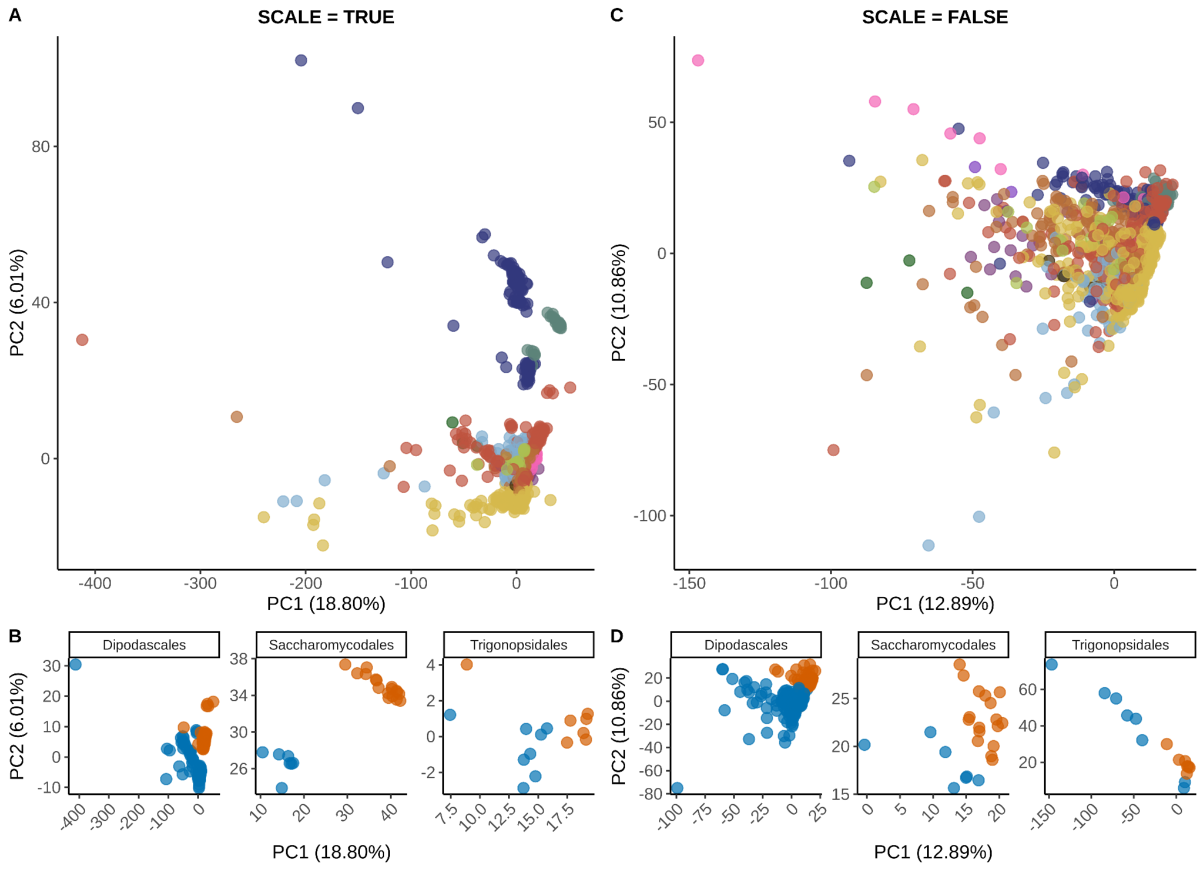

Appendix Figure S12 - PCA on copy number datasets.

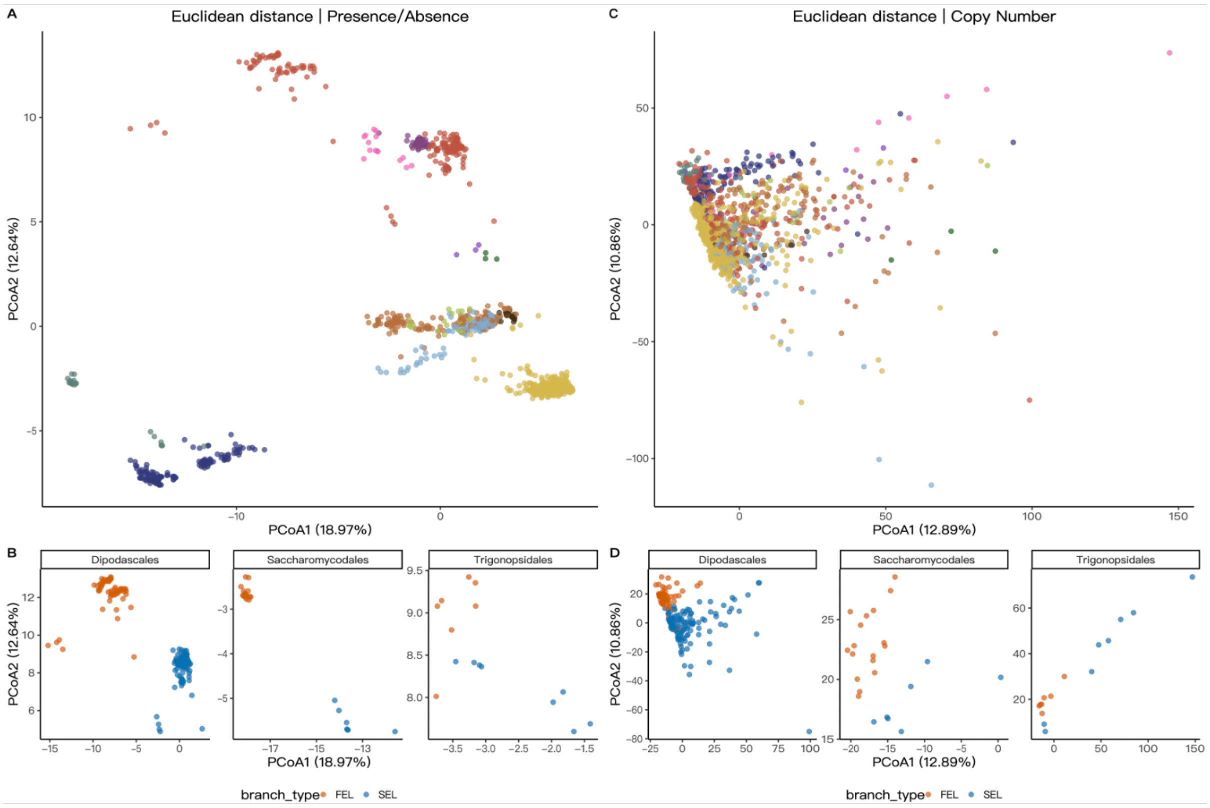

Appendix Figure S13 - PCoA on presence/absence dataset using Euclidean distance.

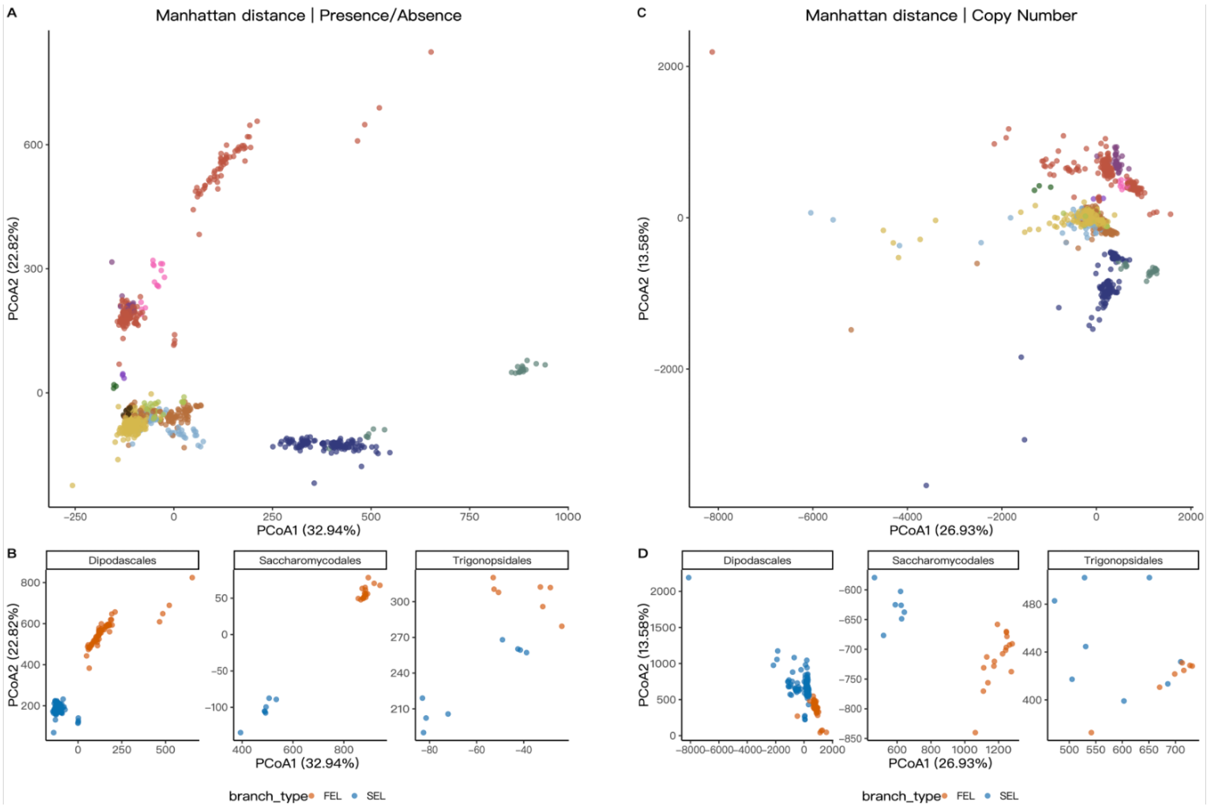

Appendix Figure S14 - PCoA on presence/absence dataset using Manhattan distance.

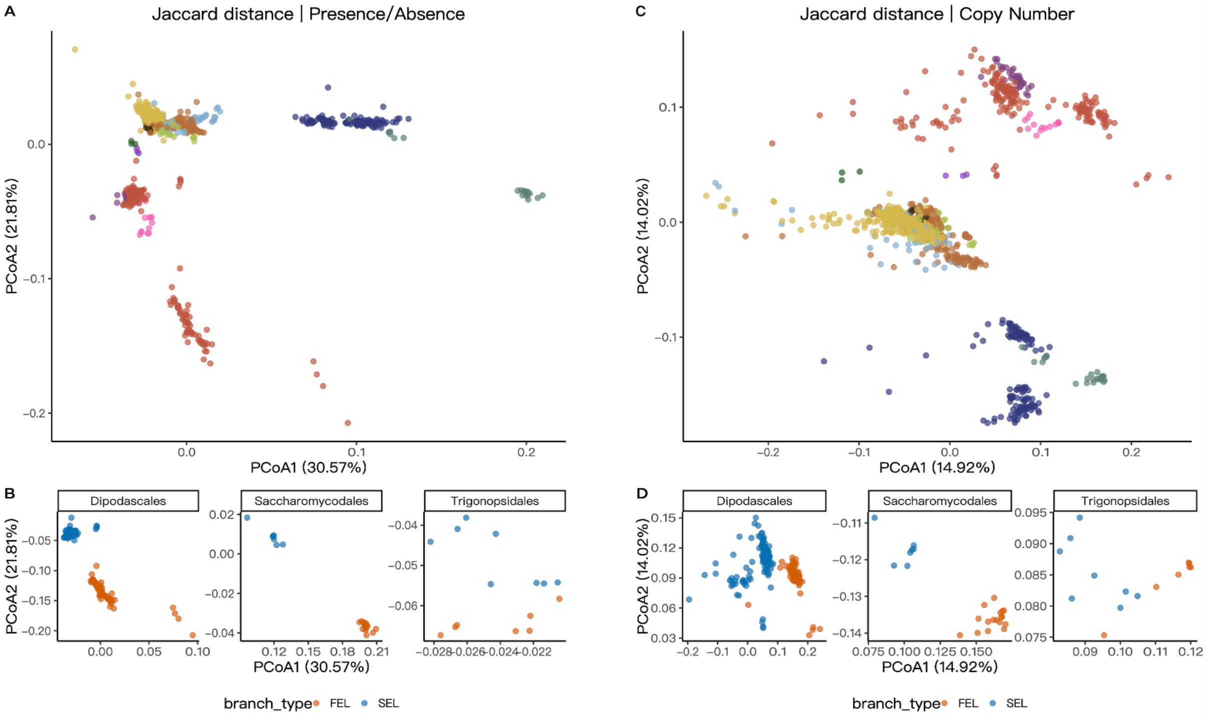

Appendix Figure S15 - PCoA on presence/absence dataset using Jaccard distance.

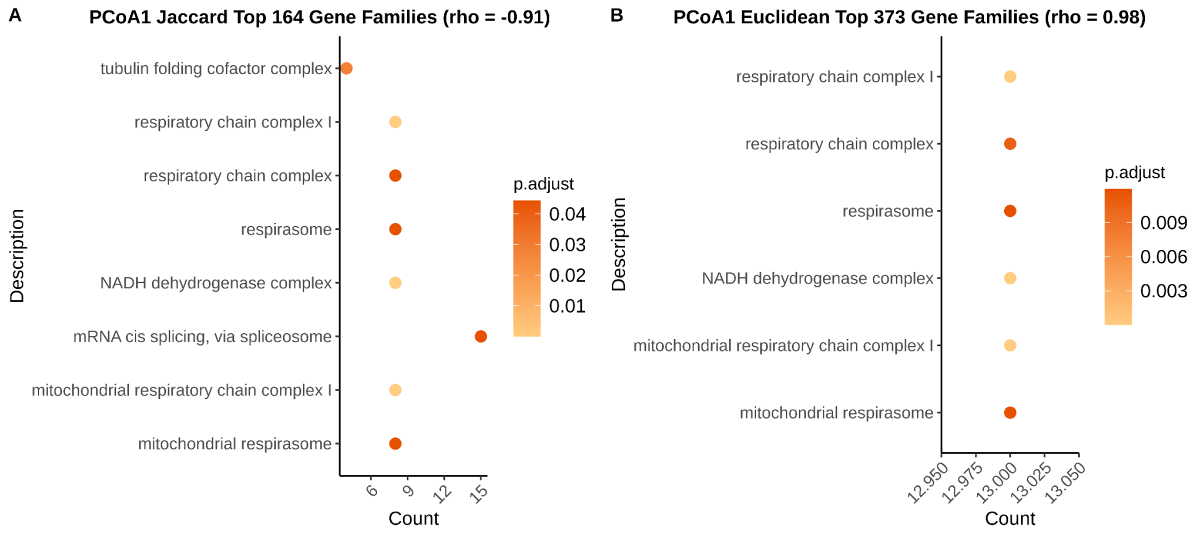

Appendix Figure S16 - Functional enrichment analysis on PCoA1 using Jaccard and Euclidean results.

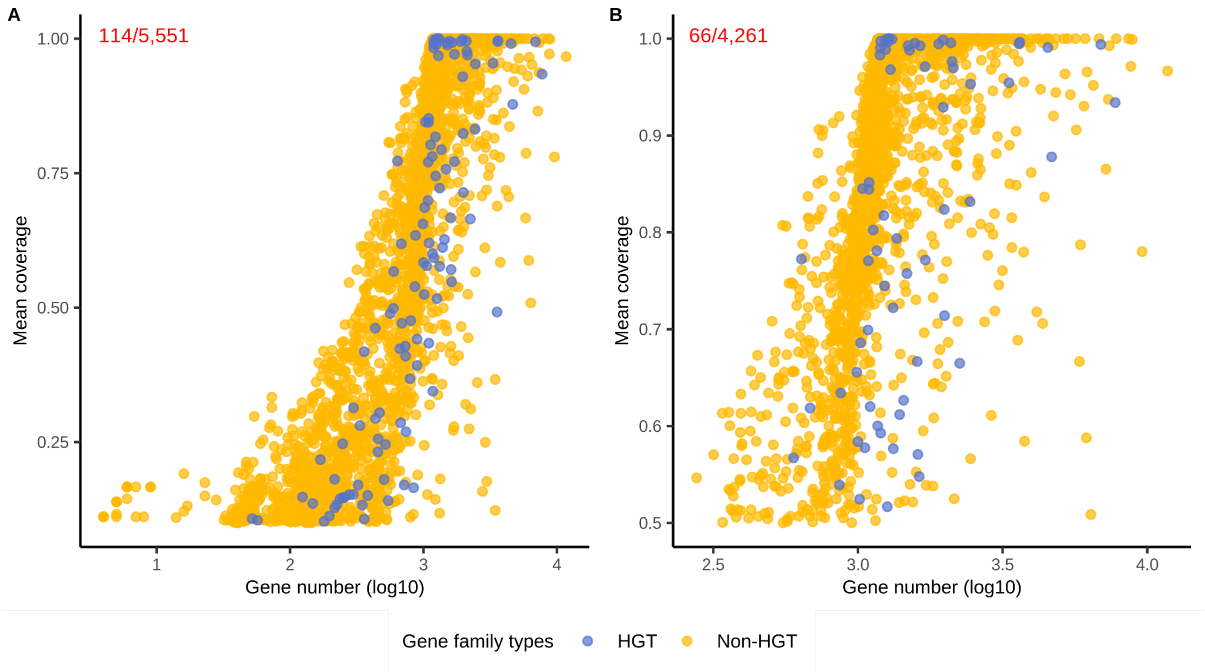

Appendix Figure S17 - Distribution of HGT genes in 0.1 and 0.5 coverage gene family datasets.

A Mean coverage indicates the average coverage of gene families across 12 orders of Saccharomycotina yeasts, with 114 out of 5,551 gene families containing HGT genes in the 0.1 coverage dataset.

B 66 out of 4,261 gene families contain HGT genes in the 0.5 coverage dataset.

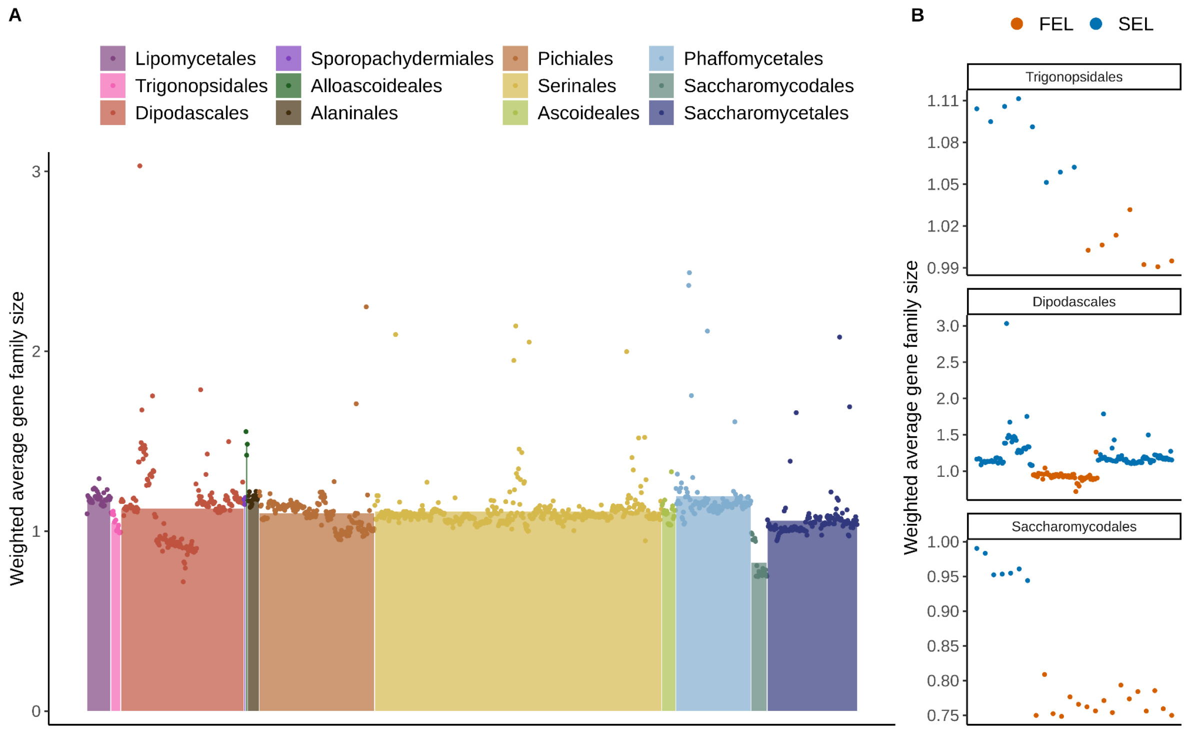

Appendix Figure S18 - Weighted average gene family size of 0.1 coverage dataset excluding HGT genes.

The arrangement of yeasts on the x-axis follows the same order as in the phylogenetic tree and aligns with Figure 2 and Figure EV2 in our manuscript.

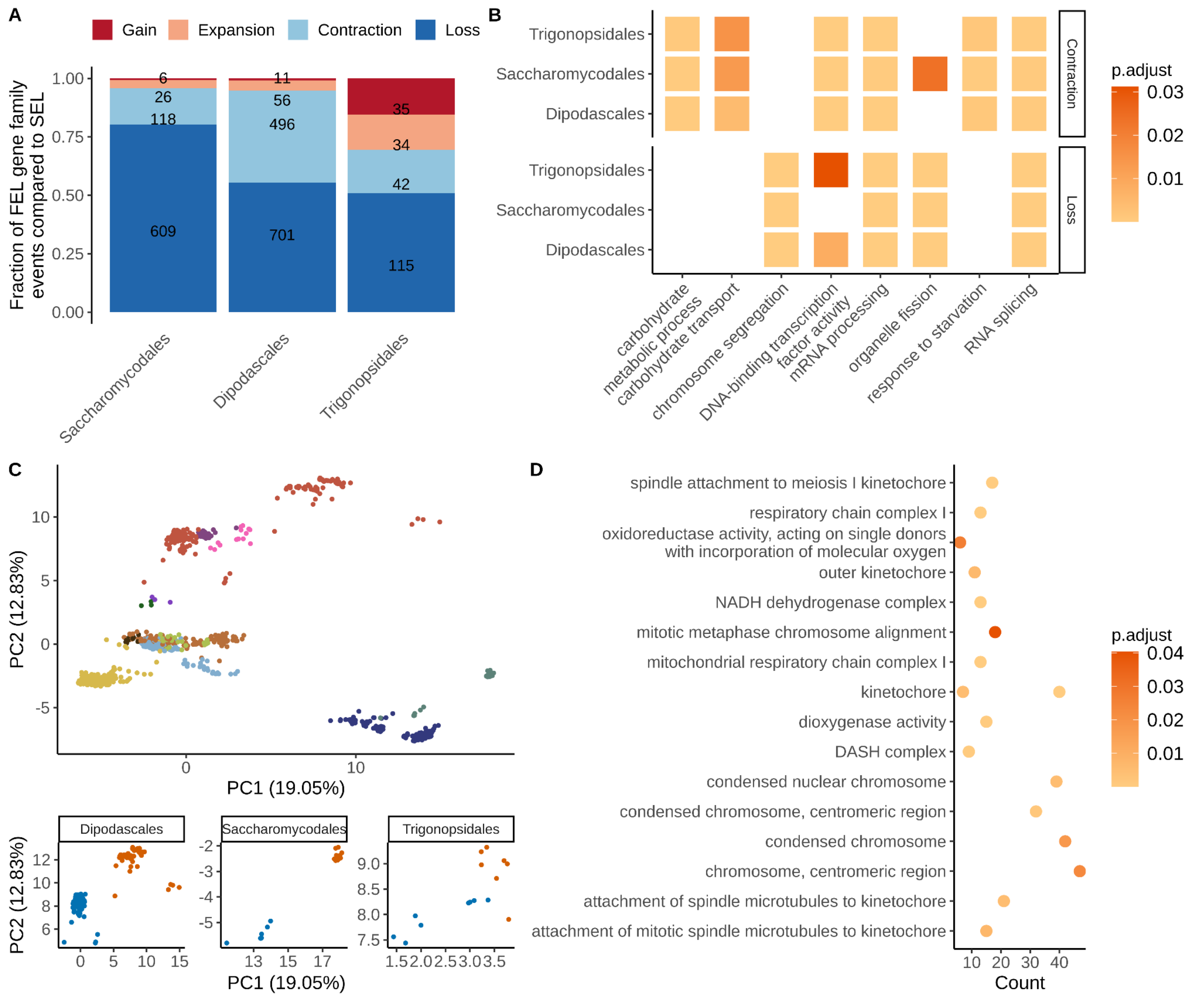

Appendix Figure S19 - Fold change PCA, and functional enrichment analyses excluding HGT genes.

Similar to Figure 3 in our manuscript, but A and B use the 0.1 coverage dataset excluding HGT genes, while C and D use the 0.5 coverage dataset excluding HGT genes.

Appendix Table S1 - Correlations between PICs of weighted average gene family size with both gene number and genome size. Spearman correlation test was applied.

Group	rho (weighted average gene family size and gene number)	p-value (weighted average gene family size and gene number)	rho (weighted average gene family size and genome size)	p-value (weighted average gene family size and genome size)
Yeast	0.8199017	0	0.5323168	0
Pezizomycotina	0.8834255	0	0.3657363	0
Animal	0.6241225	0	0.2831597	0.01016
Plant	0.9690718	0	0.1474972	0.435

Appendix Table S2 - Multimodality analyses in weighted average size and evolutionary rate within each order.

Order	p-value weighted average size	p-value evolutionay rate
Alloascoideales	1	1
Sporopachydermiales	1	1
Serinales	0.997323832	0.003668801
Phaffomycetales	0.991756123	0.935578161
Lipomycetales	0.901505761	0.159210136
Alaninales	0.827514951	0.786937806
Saccharomycetales	0.756610474	0.543129948
Trigonopsidales	0.230157621	0.00081514
Pichiales	0.215382866	0.004283324
Ascoideales	0.066956415	0.362651095
Saccharomycodales	0.01857093	0.010290885
Dipodascales	0	0.040924718

Appendix Table S3 - Gene families that experienced significant contractions or losses in Saccharomycodales and Trigonopsidales, corresponding to Dipodascales. KS test with Bonferroni correction was applied.

Orthogroup	Fold change	p.adjust	State	Order	Gene name
OG0000437	0.466666667	2.87E-05	contraction	Saccharomycodaels	*TDH3*
OG0003336	0	2.87E-05	loss	Saccharomycodaels	*ASK1*
OG0003342	0	2.87E-05	loss	Saccharomycodaels	*PRP40*
OG0003376	0	2.87E-05	loss	Saccharomycodaels	*SLU7*
OG0003405	0	2.87E-05	loss	Saccharomycodaels	*SNU23*
OG0003442	0	2.87E-05	loss	Saccharomycodaels	*SYF2*
OG0003494	0	2.87E-05	loss	Saccharomycodaels	*ISY1*
OG0003505	0	2.87E-05	loss	Saccharomycodaels	*DAM1*
OG0003525	0	0.000438078	loss	Saccharomycodaels	*CWC21*
OG0003551	0	2.87E-05	loss	Saccharomycodaels	*DAD3*
OG0003593	0	2.87E-05	loss	Saccharomycodaels	*SPC34*
OG0003704	0	0.000438078	loss	Saccharomycodaels	*SPC19*
OG0003834	0	0.003772474	loss	Saccharomycodaels	*DAD1*
OG0003886	0	2.87E-05	loss	Saccharomycodaels	*DAD4*
OG0003704	0	0.00563808	loss	Trigonopsidales	*SPC19*
OG0003834	0.142857143	0.032757959	contraction	Trigonopsidales	*DAD1*
