# Supplementary material for: Unique trajectory of gene family evolution from genomic analysis of nearly all known species in an ancient yeast lineage
Source: Mol Syst Biol. 2025 May 27;21(8):1066–89. doi: 10.1038/s44320-025-00118-0 (PMC12322030; doi:10.1038/s44320-025-00118-0)
Supplement: Supplementary file 8 — Expanded View Figures [file 44320_2025_118_MOESM8_ESM.pdf]

Expanded View Figures

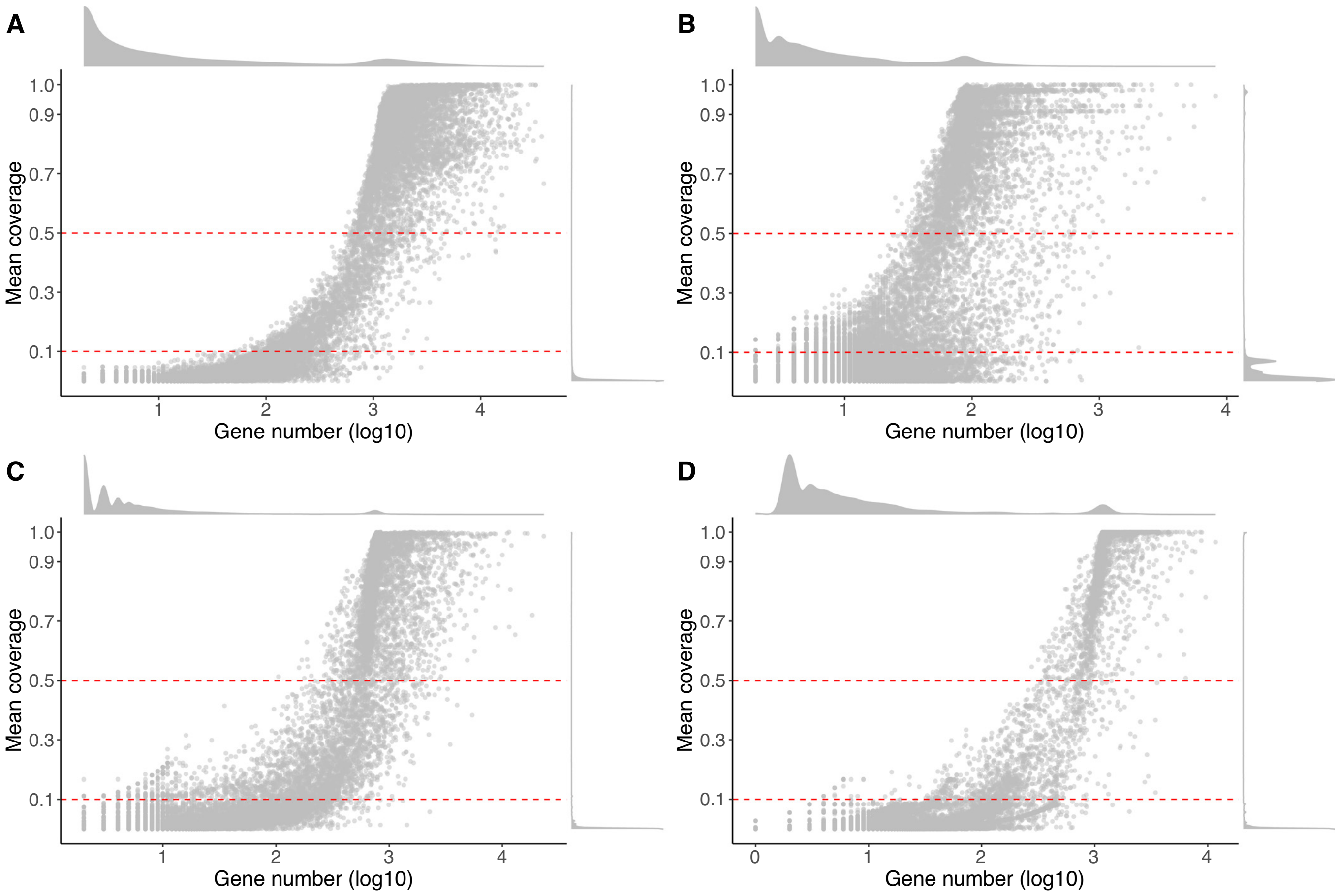

Figure EV1. Mean coverage of each gene family across 4 groups.

Mean coverage represents the average coverage of gene families across clades: plants ($n = 21$), animals ($n = 14$), Pezizomycotina ($n = 9$), and Saccharomycotina yeasts ($n = 12$). The panels are designated as follows: (**A**) plant; (**B**) animal; (**C**) Pezizomycotina; (**D**) Saccharomycotina yeast.

 © The Author(s)

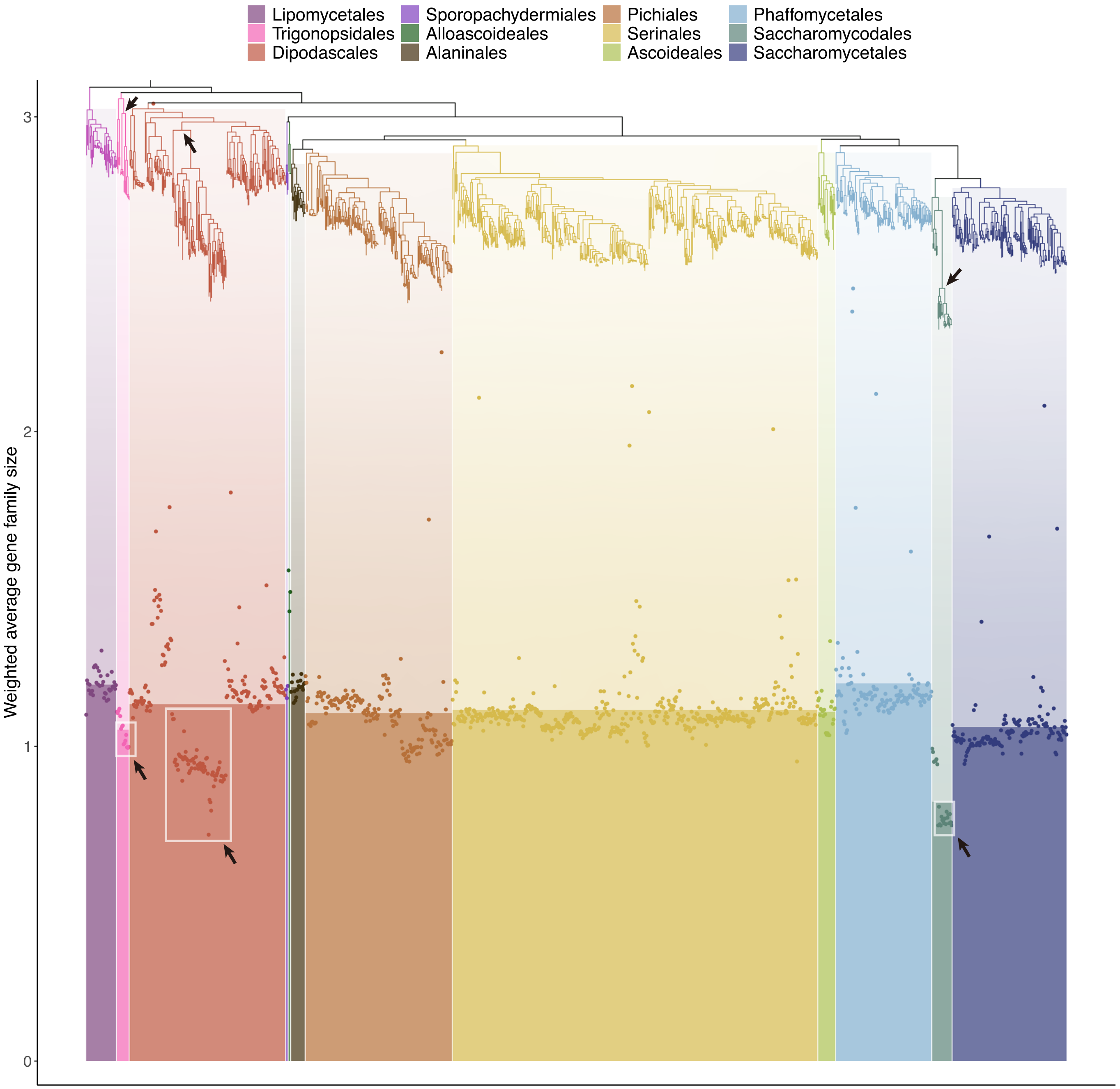

Figure EV2. Weighted average gene family sizes across 12 orders.

The scatter plot arranges yeast species in accordance with their placement on the phylogenetic tree, with the rectangle width depicting the number of genomes within each order. The darker color in each rectangle signifies the average of weighted average gene family size within that order, and the lighter color aligns the rectangle with its specific location on the phylogenetic tree. From left to right, the orders are represented as follows: Lipomycetales ($n = 36$), Trigonopsidales ($n = 15$), Dipodascales ($n = 184$), Sporopachydermiales ($n = 3$), Alloascoideales ($n = 3$), Ascoideales ($n = 21$), Alaninales ($n = 17$), Pichiales ($n = 173$), Serinales ($n = 430$), Phaffomycetales ($n = 113$), Saccharomycodales ($n = 24$), and Saccharomycetales ($n = 135$). A white box and arrow highlight the FELs in Trigonopsidales, Dipodascales, and Saccharomycodales.

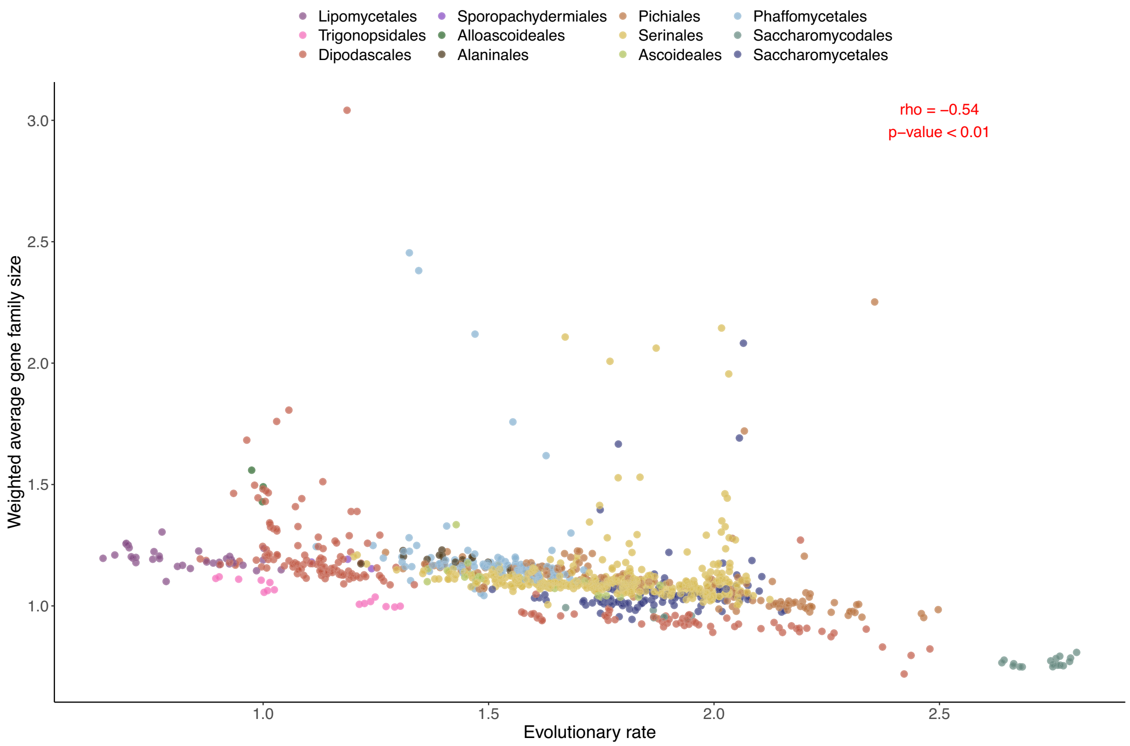

Figure EV3. Comparative analyses of weighted average gene family sizes versus evolutionary rates in FEL and SEL gene families.

The evolutionary rate was calculated using the branch length from the tip to the root in the phylogenetic tree. A Spearman test was conducted to assess the correlation between the weighted average size and evolutionary rate across 1154 yeasts (rho = −0.54, $P < 2.2 \times 10^{-16}$).

 © The Author(s)

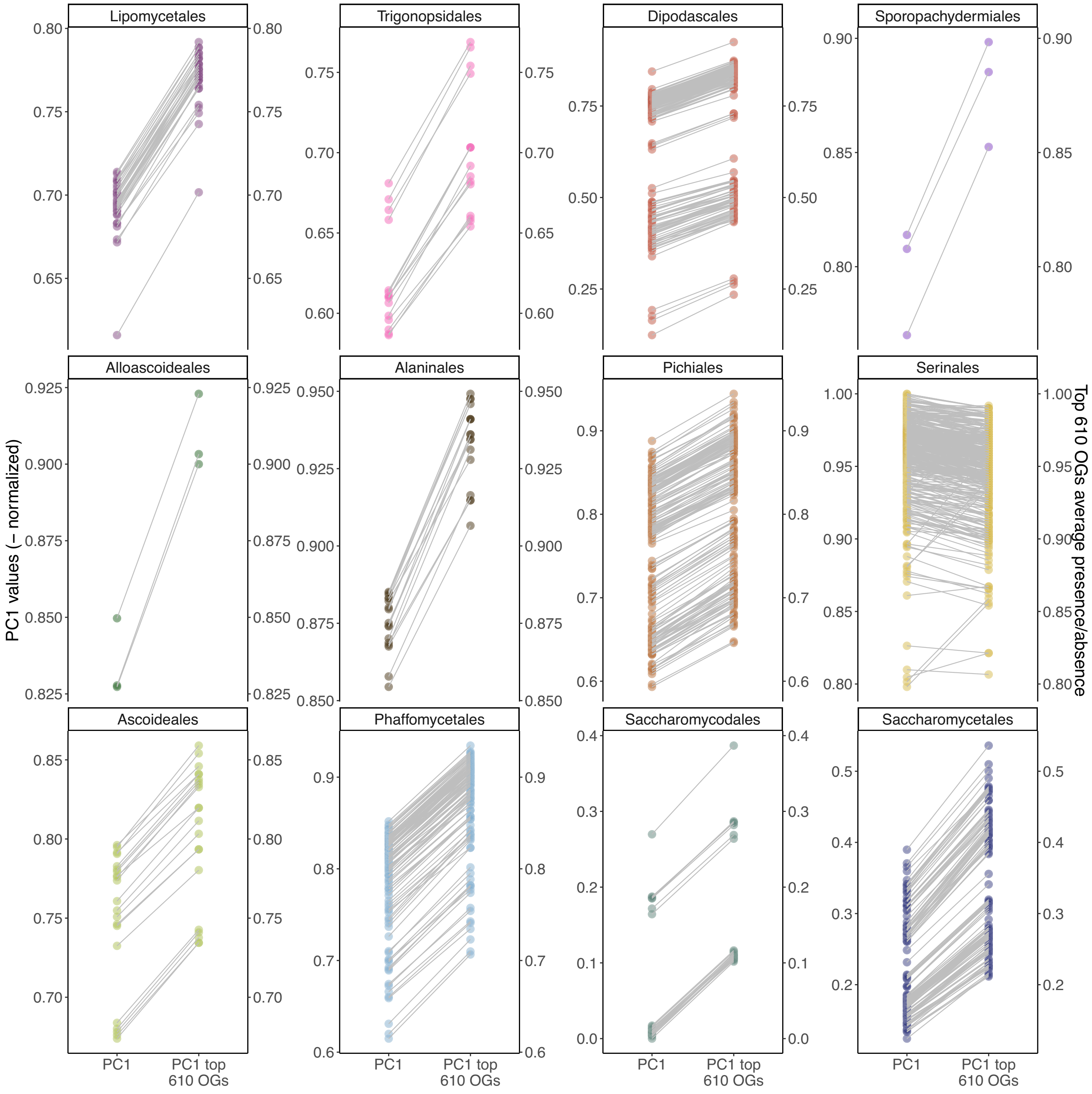

Figure EV4. Identification of 610 gene families representing PC1 in the PCA with a 0.5 coverage threshold.

The correlation analysis assessed the connection between PC1 coordinates and the average presence and absence data for the top 610 gene families, which exhibited the highest absolute correlation (rho = −0.99) via the Spearman test. PC1 coordinates were reversed and then normalized. Points on the plot represent individual yeasts, with lines connecting the same point.

© The Author(s)

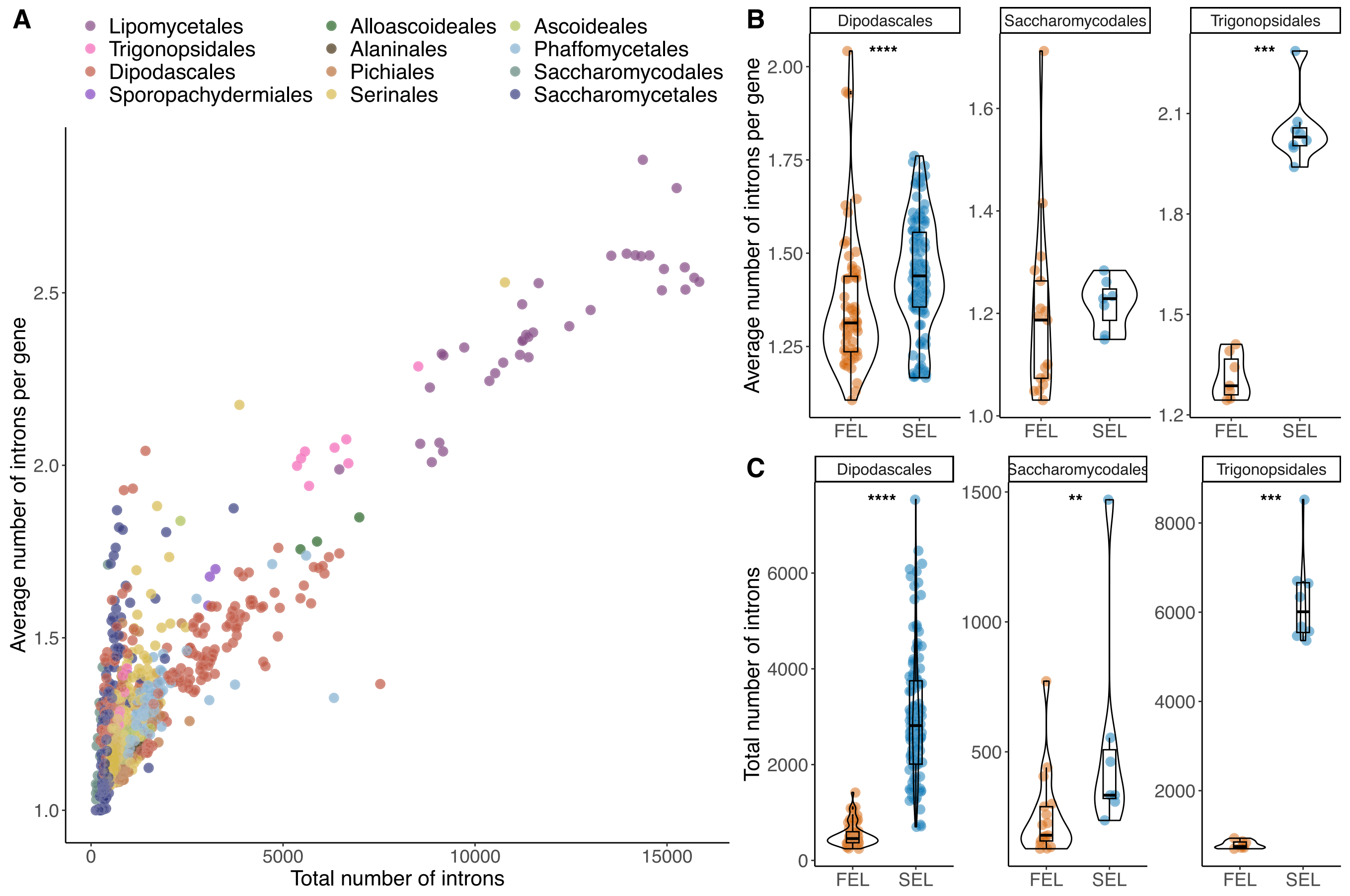

Figure EV5. Comparative analysis of intron number between FELs and SELs.

(**A**) Average intron number per gene and total number of introns across 1154 yeast species. (**B**) Comparison of average intron number per gene between FEL and SEL yeasts. Wilcoxon tests were used to determine significance, where "**" represents $P < 0.01$, "***" denotes $P < 0.001$, "****" indicates $P < 0.0001$. Exact *p*-values: Dipodascales, $P = 2.02 \times 10^{-4}$; Saccharomycodales, $P = 0.29$; Trigonopsidales $P = 3.11 \times 10^{-4}$. The center of each box plot represents the median performance, the box boundaries correspond to the upper and lower quartiles, and the whiskers extend to the 5th and 95th percentiles. The sample sizes (*n*) are as follows: for Dipodascales, FEL = 61 and SEL = 123; for Saccharomycodales, FEL = 17 and SEL = 7; for Trigonopsidales, FEL = 7 and SEL = 8. (**C**) Comparison of the total number of introns between FEL and SEL yeasts. Wilcoxon tests were used to determine significance, where "**" represents $P < 0.01$, "***" denotes $P < 0.001$, "****" indicates $P < 0.0001$. Exact *p*-values: Dipodascales, $P < 2.2 \times 10^{-16}$; Saccharomycodales, $P = 7.63 \times 10^{-3}$; Trigonopsidales $P = 3.11 \times 10^{-4}$. The center of each box plot represents the median performance, the box boundaries correspond to the upper and lower quartiles, and the whiskers extend to the 5th and 95th percentiles. The sample sizes (*n*) are as follows: for Dipodascales, FEL = 61 and SEL = 123; for Saccharomycodales, FEL = 17 and SEL = 7; for Trigonopsidales, FEL = 7 and SEL = 8.

 © The Author(s)
